# Diarrheal disease and enteric infections in LMIC communities: how big is the problem?

**DOI:** 10.1186/s40794-016-0028-7

**Published:** 2016-07-19

**Authors:** Benjamin J. J. McCormick, Dennis R. Lang

**Affiliations:** 1 Fogarty International Center, National Institutes of Health, Bethesda, MD USA; 2 Foundation for the National Institutes of Health, Bethesda, MD USA

**Keywords:** Enteric pathogens, Malnutrition, Enteropathy, Diarrhea, Cognitive development, Growth, Vaccine response, Sanitation and hygiene

## Abstract

Studies of enteric diseases have historically focused on observations of clinical diarrhea as a cause of mortality and morbidity. Emerging evidence suggests that diarrhea dramatically underestimates both exposure to enteropathogens and the long-term consequences arising from infection. High burden of pathogens in the gut, even in the absence of diarrhea, is common in infants in low and middle income countries. Continual challenge by pathogens, in conjunction with an inadequate diet stimulates an inflammatory disease that alters the structure of the gut, metabolic and immunological pathways and changes the microbiome. Both diarrhea and enteropathogen infection have been associated with reduced growth, reduced cognitive development, and reduced vaccine efficacy suggesting that the burden of diarrheal disease is dramatically underestimated.

## Background

### The importance of diarrheal disease

The importance of diarrhoeal disease to child development in low and middle income countries (LMIC) was highlighted by seminal work in the 1950s and ‘60s [1, 2]. Subsequent research over the next two decades focused on quantifying health outcomes by the frequency and severity of symptoms [3]. Interventions to interrupt transmission routes [4–6] have simultaneously greatly reduced mortality [7] and rates of diarrheal incidence [8]. Nevertheless, continuing interest in diarrhea reflects its role as the second most common cause of death in children under 5 years old [9].

**Fig. 1:**
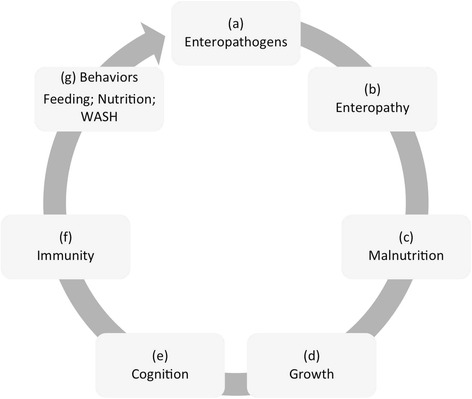
The conceptual model of the “vicious cycle of poverty”, linking enteric infections to gut dysfunction, impaired nutrient absorption, malnutrition and restricted physical and cognitive development. Stages within this cycle are described in the text

The burden of diarrhea, however, is not solely estimated by mortality. It is a proximate driver of malnutrition [10], and related to many insidious morbidities [3, 11]. A considerable body of evidence has rearranged the linear United Nations Children's Emergency Fund (UNICEF) framework [10] of malnutrition into a cyclical conceptual model that describes a ‘vicious cycle of poverty’ [11] (Fig. 1): (a) infection with enteric pathogens is associated with b) impaired gut-function [12–15] that can (c) exacerbate effects of malnutrition [16], for example, restricting appropriate processing of nutrients [17, 18] necessary for (d) physical [17–20] and (e) cognitive development [21–23] as well as (f) altering immune responses [16, 24], thereby impairing a child’s ability to resist a) recurrent infections [17, 25] and illness [26]. Scaling to a population from an individual, malnutrition as a disease associated with poverty increases (g) behaviors and environments that propagate the syndrome. It is worth noting that the language of the vicious cycle has evolved from a focus on diarrhea [27] to specifically implicating enteropathogen infection absent diarrhea [11, 28]. Advances in diagnostics are likely to provide further evidence for the role of cryptic infections in the absence of overt symptoms.

## Main text

### Diarrhea and enteropathogens

Recorded rates of diarrhea are unlikely to reflect the true prevalence of disease and thereby dramatically underestimate the untoward consequences of enteropathogen infections. In England where care is readily available, unreported diarrhea was 5.8 times higher than those that were recorded [29] and in Canada only 10 % of adults sought care [30]. Where symptoms are considered common or expected, they are less likely to be reported.

Early research focused on diarrheal symptoms on the presumption that childhood diarrhea in LMIC settings was the product of underlying infection [31]. Identification of which pathogen was causing diarrhea was just awaiting development of methods of detection. In the 1960s recovering a pathogen from diarrheal stool was seldom possible in more than 40 % of cases. For recent studies the challenge has been to ascribe causation between the many pathogens detected, either in the same stool or given the high rates of detection in the absence of diarrhea. In the MAL-ED study 76.9 % diarrheal stools and 64.9 % of non-diarrheal stools had at least one pathogen detected and 41.0 % and 29.0 %, respectively, had at least two [32]. Similarly in the Global Enteric Multicenter Study (GEMS), though examining moderate to severe diarrhea rather than the prospective community cohort of MAL-ED, found a comparable 83 % of cases and 72 % of controls had at least one pathogen, with two or more identified in 45 % cases and 31 % controls [33]. Rates of detection regardless of coincident symptoms are only likely to rise as more sophisticated technology (e.g. qPCR) enables both increased rates of detection as well as quantitation of pathogen burden [34, 35].

The attribution of diarrhea to specific pathogens is more challenging when they are coincident, although estimates appear remarkably similar to those of the 1960s when a single pathogen was detected in stool. In GEMS, an average of 44 % cases of moderate-to-severe diarrhea in infants and 47 % of cases in toddlers were attributed to pathogens. MAL-ED attributed 19.1 % and 33.1 % of diarrheal episodes in the first and second year of life respectively to enteropathogens. Even if pathogens appear less causally linked to diarrhea than previously expected, the high prevalence of these organisms in the absence of diarrhea raises the question of whether or not these organisms are causing more covert damage?

### Enteropathogens and enteric disease

Histopathogical studies, dating back to the 1960s, of intestinal biopsy samples revealed physiological and functional changes in the gut - villus atrophy and crypt hyperplasia [36–38] that have been attributed to living in an environment where repeated enteropathogen infections were common [39]. In an effort to find non-invasive diagnostics and the absence of overt pathognomonic symptoms, a growing collection of biomarkers [40, 41] are emerging that can detect reduced absorptive capacity [39, 42], increased permeability [43] and chronic intestinal inflammation [44, 45] that have become indicative of an inflammatory condition induced by enteropathogens called environmental enteropathy (EE) [46, 47].

Many studies have examined biomarkers of EE and found associations between EE and malnutrition. The ratio of lactulose to mannitol, as an indicator of absorptive capacity and/or breaches of the mucosal barrier has explained as much as 42.9 % of linear and 38.9 % of weight variability [43] though not consistently in other populations [48–50]. This variability may be attributed to differences in testing procedure, test sensitivity [51, 52], or to variability in how populations process the two sugars to drive the ratio [39] and their individual analysis (sometimes categorized rather than continuous). Another individual biomarker of inflammation, REG1B, explained around 1.5 % of linear growth variation [53]. However, using a broader spectrum of EE biomarkers to capture multiple aspects of the syndrome achieved a description of 46.3 % of linear growth variation [24].

Exciting new understanding is emerging from mouse models that offer not just confirmation of EE, but also causal explanation. Mice fed a malnourished diet to mimic diets of children in Bangladesh exhibited 30 % reduction in weight gain, however it was only when malnourished mice were additionally exposed to gut bacteria that they developed similar gut lesions found by histopathology [16]. The addition of a *Bacteroidales* and *E. coli* cocktail exacerbated much of the intestinal damage of moderate malnutrition including altering bacterial colonisation, inflammation and immunity to other enteropathogens. However, enteropathogens importantly interact with the commensal microbiome [54, 55], which is shaped by diet [56–58]. In malnourished children the gut microbiota remains immature and its composition and colonisation patterns fail to mature with age as the microbiota does in well-nourished children. Diet-induced changes to the gut environment along with frequent exposure to new microbes result in demonstrable changes in immune and metabolic pathways [16, 59, 60]. Relating the pathways found within mouse models to observational studies in humans using biomarkers is proving more difficult, but EE does provide a mechanistic link between enteropathogen exposure and undernutrition to growth and development.

### Infection and growth

Because it is easily identified, most studies have relied on diarrheal symptoms as an indication of enteropathogen infection. Diarrhea has long been associated with growth shortfall; 5, 11 and 20 % of reduced linear growth in Mexico, Guatemala and Bangladesh respectively [61–63]. A pooled analysis of nine studies found consistent associations between diarrhea and stunting with 25 % of stunting prevalence at 2 years old attributed to children with ≥5 diarrhoeal episodes, or alternatively 18 % of stunting attributed to children who had diarrhoea on ≥2 % of days from birth to 24 months [64]. Improving sanitation and hygiene has been predicted to reduce stunting by 6 % [65] and even reduce all-cause mortality by 4 % [66].

Quantifying the contribution of individual pathogens to growth shortfalls has been less frequently examined. *E. coli* and *Shigella* were associated with decreased weight and length respectively in Bangladesh [63] and in addition to these pathogens, infection with *Campylobacter* was associated with reduced linear growth in Peru [20]. Pathogens, for example *Cryptosporidium* and *Campylobacter*, in diarrhea are associated with greater growth deficits than those with no symptoms [67, 68] though the prevalence of the latter is likely to be more common.

Whilst pathogens and diarrhea have been associated with reduced growth, the effects can be reversible. For example, weight loss following infection with *Shigella* could be reversed with improved diet [69]. In some cases growth retardation caused by transient infection can recover without intervention [19]. Repeated infections diminish the ability to achieve catch-up growth to compensate for growth shortfall [70, 71].

### Infection and oral polio vaccine

Malnutrition, has been implicated as a reason for the disappointing efficacy of oral vaccines in developing countries [72–74]. Diarrhea at the time of oral polio vaccine (OPV) reduces the odds of seroconversion to 0.68 [75], and malnutrition reduced OPV titers by half [76]. Similarly, rotavirus vaccine efficacy, important because of the association between rotavirus and diarrhea [32, 33], is only 51 % in developing regions [77] and illness (both diarrheal and respiratory) both during or after measles vaccination can result in reduced titers [78].

Individual pathogens, in addition to contributing to overt illness and general malnutrition, can impair vaccine efficacy. Plasma phagocytic activity, for example is reduced by enterotoxigenic *E. coli*, which additionally reduced complement C3 levels by 50 % [79]. Ascaris infections and small bowel bacterial overgrowth (SBBO) reduced CVD 103-HgR cholera vaccine response [80, 81]. SBBO is associated with gut inflammation [82], an important component of EE which can result in modulation of immune pathways [16, 24] that can, along with gut microbiota [54, 83], reduce the efficacy of vaccines. Mechanisms can include production of small molecules that are cross-reactive with oral vaccines and increased mucosal lymphoctyes and plasmacytes, and notably T-cells [16, 44]. Levine [74] suggests two explanations, one in which EE elicits a chronically pro-inflammatory state that protects against repeated infection, while at the same time diminishing response to orally delivered live vaccines. Alternatively, a second possibility is that active immune responses are dampened as the gut microbiota and host immunity is modified so that pathogens are tolerated rather than cleared [84, 85].

### Infection and cognitive development

As diarrheal disease disrupts nutrient processing and metabolic pathways, one postulated consequence of the vicious cycle is that repeated infections impair cognitive development. Correlative evidence gives some support to this idea, with numerous studies demonstrating that stunted children perform less well in early assessment of cognitive performance and that early tests of cognitive development are predictive of school readiness and achievement [86]. More specifically, studies have observed that higher rates of diarrhea [23, 87] and some pathogens [21, 88–90] delay school-readiness though results are disputable when other correlated factors are accounted for, such as underlying poverty or malnutrition [89, 91].

The consequence of impaired cognitive development, most of which has associated with poverty is estimated to reduce adult income by 5.9 %, and, when combined with stunting, reduces income by 30.1 % [86]. This is largely the result of lost years of primary schooling, through delayed school readiness, high drop-out rates or reduced learning whilst at school [92], each of which is estimated to account for a drop of 6.8 to 10.6 % in adult wages [93]. With reduced wages, the vicious cycle is perpetuated, with negative effects on future generations and populations.

## Conclusions

A mounting body of evidence suggests that the burden of diarrhea is much greater than the high reported number of childhood deaths each year. The insidious morbidity ascribed to diarrheal disease is more appropriately attributed to enteric infection.

**Fig. 2:**
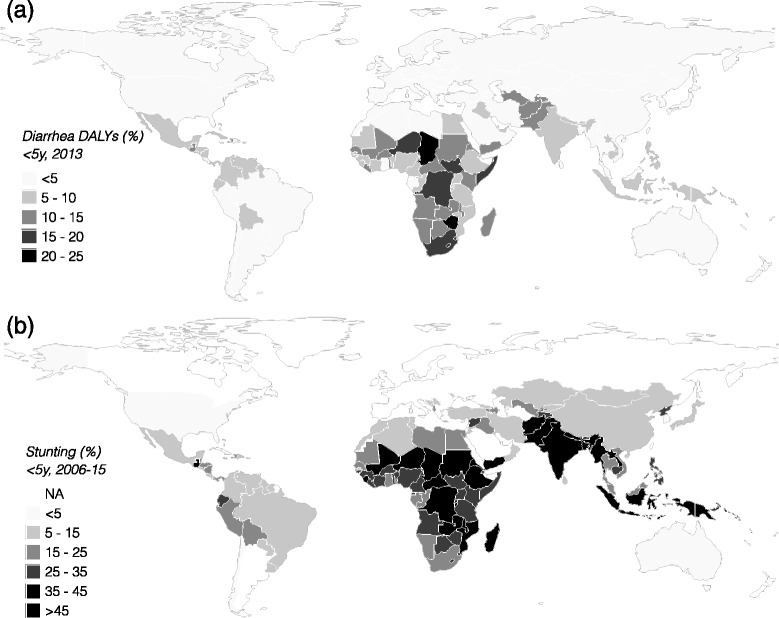
**a**) The percentage of DALYs in children under 5 years old due to diarrheal disease based on the 2013 Global Burden of Diarrhea estimates [95]; **b**) the percentage of children stunted based on data collated by UNICEF, WHO, World Bank showing the most recent national data available for each country (ranging from 2006 to 2015) [96]

Guerrant et al. [94] offered a first attempt to revise estimates of diarrheal disease Disability Adjusted Life Years (DALYs) (Fig. 2). Rather than base 95 % of the burden on mortality, they accounted for long-term consequences and suggested a conservative doubling of the disease burden. As further data become available that can better identify and quantify long-term health effects of asymptomatic enteric infection, this estimate is likely to be refined, and will almost certainly be increased, for example, accounting for at least some of burden of stunting, which is substantially greater than reflected by current DALY estimates due to diarrhea per se (Fig. 2).

By explicitly recognising the importance of exposure to pathogens, singly or in combination, and not being guided solely by the symptom of diarrhea, targeted efforts to reduce the compounded damage due to inadequate diet and infection may yield greater success than reducing either separately. It suggests that increased emphasis should be placed on improving access to clean water, adequate sanitation and hygiene (WASH) methodologies that attempt to break transmission routes that are common to many pathogens along side targeted vaccination against high priority pathogens.

## Abbreviations

DALYs: Disability Adjusted Life Years
EE: environmental enteropathy
GEMS: Global Enteric Multicenter Study
LMIC: low and middle income countries
MAL-ED: The Etiology Risk Factors and Interactions of Enteric Infections and Malnutrition and the Consequences for Child Health and Development
SBBO: small bowel bacterial overgrowth
UNICEF: United Nations Children's Emergency Fund
WASH: Water Adequate Sanitation and Hygiene
